# Investigation of Oral Toxicity Mechanisms of Low-Molecular-Weight Polycyclic Aromatic Hydrocarbons and Probiotic Interventions Under the Framework of the “Gut–Lung Axis”

**DOI:** 10.3390/foods15193446

**Published:** 2026-09-27

**Authors:** Yixuan Hu, Zhixing Chen, Dan Luo, Zhenqiang Tang, Shiyao Jiang, Jiebin Pan, Yanzong Zhao, Junling Wang, Yixing Pan

**Affiliations:** 1 School of Public Health, Lanzhou University, Lanzhou 730000, China; 2 The Second Hospital & Clinical Medical School, Lanzhou University, 222 Tianshui South Road, Lanzhou 730000, China; 3 School of Public Health, Huazhong University of Science and Technology, Wuhan 430030, China; 4 School of Stomatology, Lanzhou University, Lanzhou 730000, China

**Keywords:** low-molecular-weight polycyclic aromatic hydrocarbons, gut–lung axis, pulmonary injury, gut microbiota, probiotic intervention

## Abstract

Low-molecular-weight polycyclic aromatic hydrocarbons (LMW-PAHs), as ubiquitous contaminants in the environment and food processing, have attracted growing concern due to their systemic health hazards through oral exposure. Recent studies have demonstrated that the toxic effects of LMW-PAHs extend beyond the direct damage caused by inhalation exposure. They can induce pathological changes in distal organs by disrupting the bidirectional communication system known as the “gut–lung axis”. This review summarizes the major sources and health risks of LMW-PAHs and elucidates the communication mechanisms underlying the gut–lung axis, which comprises a bidirectional regulatory network involving gut microbial metabolites, circulating inflammatory factors, immune cell migration, and neuroendocrine signaling. Furthermore, the review explores the core mechanisms by which oral exposure to LMW-PAHs leads to pulmonary injury: primarily through triggering gut microbiota (GM) dysbiosis, compromising intestinal barrier integrity, and inducing intestinal inflammation, which collectively propagate lung injury via the “gut–lung axis”. In response to these toxic pathways, we propose probiotic intervention strategies that may antagonize the gut–lung-axis-mediated toxicity of LMW-PAHs through multiple mechanisms, including modulation of gut microbiota composition, reinforcement of intestinal barrier integrity, systemic immune regulation, and production of beneficial metabolites such as short-chain fatty acids (SCFAs). Finally, this review discusses the application prospects and existing challenges of probiotic interventions, aiming to provide a solid theoretical foundation and novel perspective for probiotic resource development and application based on the “gut–lung axis”.

## 1. Introduction

With the rapid advancement of industrialization and urbanization, polycyclic aromatic hydrocarbons (PAHs)—a class of ubiquitous and chemically stable persistent organic pollutants—have become a major global environmental and public health concern. PAHs are aromatic hydrocarbons containing two or more fused benzene rings. Based on the number of rings and molecular weight, PAHs containing four or more rings are categorized as high-molecular-weight polycyclic aromatic hydrocarbons (HMW-PAHs), while those containing two to three rings (such as naphthalene, anthracene, and phenanthrene) are defined as low-molecular-weight polycyclic aromatic hydrocarbons (LMW-PAHs) [1]. These compounds can enter the human body through multiple routes, including inhalation, dietary intake, and dermal contact. Compared with HMW-PAHs, LMW-PAHs possess lower molecular weights and typically exhibit higher volatility, water solubility, and bioavailability [2]. Consequently, they display enhanced transfer ability, bioaccumulation, and biomagnification in aquatic and food chains, thereby increasing the likelihood of human exposure through contaminated food and water [3]. In recent years, growing attention has been paid to the impact of LMW-PAHs on respiratory health. Traditional research has predominantly focused on the strong carcinogenicity of HMW-PAHs and their mechanisms of inhalation-induced oxidative stress, inflammatory responses, and apoptosis [4]. However, this focus has partly underestimated the systemic health risks posed by LMW-PAHs, particularly those associated with oral exposure. Emerging evidence suggests that oral exposure to LMW-PAHs can indirectly induce pulmonary injury by disrupting the balance of the “gut–lung axis” [5].

The emergence and deepening understanding of the “gut–lung axis” concept have provided a novel perspective for interpreting the indirect effects of environmental pollutants on distant organs. The “gut–lung axis” represents a bidirectional regulatory mechanism between the intestine and lungs mediated through microbial and immune interactions. It comprises four main aspects: pulmonary diseases can alter gut microbiota (GM) composition; changes in GM can influence pulmonary diseases; intestinal diseases can modify lung microbiota; regulating lung microbiota can, in turn, affect intestinal diseases [6]. Among these, alterations in GM structure exert a particularly critical impact on lung health [7], and maintaining its microbial homeostasis is essential for sustaining normal pulmonary function.

In daily life, humans may develop GM dysbiosis by consuming food or water contaminated with PAHs, which can subsequently lead to pulmonary injury via the gut–lung axis [8]. Evidence suggests that PAH exposure may aberrantly activate the aryl hydrocarbon receptor (AhR), thereby interfering with normal AhR activation by tryptophan metabolites such as indoles. This dysregulation results in gut microbial imbalance, intestinal inflammation, and disruption of the intestinal barrier, ultimately affecting the lungs through the “gut–lung axis” [9], but research on the mechanisms of oral exposure to LMW-PAHs remains limited. Therefore, elucidating how LMW-PAHs disrupt “gut–lung axis” homeostasis to induce pulmonary injury has become a cutting-edge scientific issue in the intersection of environmental toxicology and respiratory medicine.

Confronted with this challenge, identifying safe and effective intervention strategies is of great significance. Probiotics, as viable microbial preparations, can modulate GM composition, strengthen the intestinal barrier, and exert systemic anti-inflammatory effects [10]. Recent studies have demonstrated the protective potential of probiotics in various pulmonary diseases, including chronic obstructive pulmonary disease (COPD) [11], acute lung injury (ALI) [12], and asthma [13]. The core value of probiotics lies in their natural affinity with the gut ecosystem. Rather than directly neutralizing or degrading PAHs, probiotics act as ecological modulators that remodel a healthy intestinal microenvironment through multiple mechanisms [14]. Their benefits include, but are not limited to, competitively inhibiting pathogenic bacterial colonization to regulate dysbiotic microbiota; reinforcing the intestinal barrier by enhancing tight junction protein expression and stimulating mucus secretion to prevent “leaky gut” and the translocation of toxic substances; and modulating host immune responses—such as balancing regulatory T cells (Treg) and T helper 17 (Th17) cell ratios and promoting the production of the anti-inflammatory cytokine interleukin-10 (IL-10)—to mitigate the local and systemic inflammatory storm triggered by LMW-PAHs [15]. More importantly, recent findings have unveiled deeper mechanisms of probiotic action: the bioactive metabolites generated by probiotics, including short-chain fatty acids (SCFAs), bacteriocins, and palmitic acid, can regulate the GM and alleviate PAH-induced pulmonary injury through the “gut–lung axis”. Thus, building upon the pivotal role of probiotics in maintaining gut homeostasis and systemic immunity and their close association with the “gut–lung axis” communication mechanism, this study aims to elucidate how oral exposure to LMW-PAHs disrupts gut microbiota and causes pulmonary injury via the “gut–lung axis”, and to explore probiotic-mediated mechanisms for mitigating LMW-PAHs–induced lung toxicity as well as their potential as functional food components. Literature for this review was identified by searching PubMed using combinations of the following terms: “polycyclic aromatic hydrocarbons”, “low-molecular-weight PAHs”, “gut microbiota”, “gut–lung axis”, “intestinal barrier”, “probiotics”, “short-chain fatty acids”, and “lung injury”. Reference lists of retrieved articles were also screened to identify additional relevant studies. Articles were selected based on relevance to the review’s focus—the oral toxicity of LMW-PAHs and probiotic intervention via the gut–lung axis—with priority given to recent peer-reviewed original studies and reviews. Because this is a narrative review, no formal quality assessment or quantitative synthesis was performed.

## 2. Sources of LMW-PAHs and Their Health Impacts

### 2.1. Sources and Exposure Pathways of LMW-PAHs

LMW-PAHs are pervasive environmental pollutants with diverse sources and multiple exposure routes, posing potential risks to human health and ecosystems [16]. Food represents the major exposure route for nonsmokers. LMW-PAHs are generated during food processing methods such as frying, baking, smoking, and salting and can be found in processed meat products [17]. Liquefied fat drips onto heat sources (particularly open flames), where high temperatures promote aromatization and dehydrogenation-cyclization of unsaturated hydrocarbons in oils, favoring the formation of LMW-PAHs. These compounds may then enter the human body via food intake [18]. PAHs are commonly detected in surface waters, and consuming contaminated drinking water constitutes another route of exposure [19]. Similarly, crops and aquatic organisms growing in polluted water can absorb PAHs through their root systems and accumulate them via food-chain bioaccumulation. Consequently, agricultural and aquatic products often serve as carriers of LMW-PAHs [20]. LMW-PAHs can also accumulate in food via environmental deposition; for instance, cigarette smoke, wood combustion, and automobile exhaust emissions deposit PAHs onto fruits, vegetables, and fish [3]. Moreover, seafood collected from oil-spill-affected regions has been found to contain elevated levels of LMW-PAHs [16]. In addition, incomplete combustion of organic materials, petroleum refining, vehicular exhaust emissions, petrochemical industries, forest fires, and volcanic activity release exhaust gases that introduce LMW-PAHs into the atmosphere [16]. Accordingly, LMW-PAHs may exist in gaseous form or adsorb onto Particulate Matter 2.5 (PM2.5), rendering inhalation a significant exposure route for urban residents [21]. Furthermore, PAHs present in sediments and sludge also pose health risks through dermal contact and accidental ingestion [22]. Occupational exposure is particularly relevant in industrial environments, where workers may come into contact with oil mist or materials containing LMW-PAHs through the skin [23]. To conclude, LMW-PAHs originate from food and environmental sources, enter the human body via food intake and air inhalation, and induce multiple adverse health hazards, including genotoxicity, neurodevelopmental toxicity, immunotoxicity, and endocrine disruption, as well as digestive and respiratory system damage (Figure 1). Naphthalene, anthracene, phenanthrene and fluorene are representative LMW-PAHs; they are mainly derived from fossil fuels, industrial chemicals and combustion by-products, with exposure routes involving ingestion, inhalation and dermal contact, and can cause multi-organ injuries such as respiratory, reproductive, immune and neurological damage, accompanied by genotoxicity and carcinogenic risks (Table 1).

### 2.2. Effects of LMW-PAHs on the Gut

GM comprises more than 1500 species, with *Bacteroidetes* and *Firmicutes* being the predominant phyla that together constitute approximately 90% of the total microbial population in humans. These microorganisms perform numerous physiological functions in the intestine, including defense against pathogenic invasion, enhancement of immune function, and regulation of host metabolism [28]. Exposure to LMW-PAHs has been shown to be closely associated with alterations in gut microbiota composition. For instance, studies have shown that mice administered LMW-PAHs exhibited a decline in *Firmicutes* abundance and an increase in *Bacteroidetes* abundance, suggesting that *Firmicutes* are relatively more sensitive to LMW-PAH toxicity, whereas *Bacteroidetes* show greater resistance [29]. Another study revealed dose-dependent changes in GM composition: Compared with the control group, the middle dose group showed an increased abundance of *Acidobacteriota* and *Chloroflexi*, while the high dose group prominently elevated *Lactobacillus* levels [8]. In general, LMW-PAH exposure decreases species richness of GM to some extent, and the abundance of each bacterial community will also change, leading to dysbiosis that can impair immune and digestive functions. The intestinal barrier comprises a mechanical barrier (such as intestinal epithelial cells and tight junctions) and a chemical barrier mainly formed by the mucus layer; additionally, immune cells and vascular endothelial structures within the lamina propria provide protection against pathogenic invasion [30]. Animal studies have demonstrated that exposure to phenanthrene and fluorene damages colonic epithelial integrity in rats, increasing crypt spacing and reducing the expression of intestinal tight junction proteins [8].

The GM produces SCFAs as the primary energy substrates for intestinal epithelial cells, and these metabolites can be transported to the liver to participate in gluconeogenic processes. Consequently, GM dysbiosis has been associated with obesity. GM also generates trimethylamine (TMA) and bile acids; TMA has been implicated in cardiovascular injury, while bile acids act as signaling molecules that modulate glucose and insulin tolerance [31]. Additionally, LMW-PAHs can induce intestinal inflammation. Some research using H&E staining has revealed infiltration of inflammatory cells into colon mucosa, reduced goblet cell counts, and aggregation of centrioles following LMW-PAH exposure, confirming the presence of intestinal inflammation [29]. Regarding carcinogenicity, although most studies have focused on HMW-PAHs, LMW-PAHs may also promote tumorigenesis. Experimental evidence shows that LMW-PAHs can induce colorectal tumor formation and intestinal bleeding [32].

### 2.3. Effects of LMW-PAHs on the Lung

While oral exposure represents the primary route of LMW-PAHs entry into the human body, inhalation exposure induces more evident pulmonary damage. Studies suggest that LMW-PAHs can cause lung tissue injury, decline in pulmonary function, and respiratory infections and may contribute to the development of asthma, COPD, and even lung cancer [10]. Another investigation indicated that the increase in the concentration of LMW-PAHs in the air correlates with increased outpatient visits for respiratory diseases [33], suggesting multifaceted adverse effects on lung health. Among vulnerable populations, pregnant women exposed to PAHs have been shown to have a higher risk of preterm delivery, while premature infants exposed to high-oxygen environments may subsequently develop bronchopulmonary dysplasia [34]. This article will conduct a comprehensive and in-depth analysis of lung injury caused by oral exposure to LMW-PAHs and its potential mechanisms and further clarify the key regulatory role of the “gut-lung axis” in this process.

### 2.4. Other Health Effects of LMW-PAHs

LMW-PAHs exhibit potential genotoxicity and carcinogenicity [35], as well as teratogenic, neurotoxic, immunotoxic [36], and endocrine toxicity [37]. Multiple studies have demonstrated that 32 different PAHs, including benzo[a]fluoranthene, show strong genotoxicity in the SOS Chromosome test using *Escherichia coli* PQ37 [38]. Experiments conducted in vitro also demonstrated that polycyclic aromatic hydrocarbons can be metabolically activated through cytochrome P450 enzymes and aldehyde-ketone reductase, thereby forming DNA adducts and causing DNA damage [39]. LMW-PAHs can also induce neurological defects, such as neurological deficits, memory disorders and cognitive dysfunction, and neurodevelopmental toxicity [40]. For instance, the LMW-PAH phenanthrene exhibits strong immunotoxicity. It has been reported that phenanthrene exposure significantly decreases splenic lymphocyte percentages and reduces serum IgG levels by 22.2% in the high-dose group [41]. Additionally, the estrogenic effects of LMW-PAHs, particularly under high-exposure conditions, may cause endocrine disruption in target organs [42]. Recent research further suggests that LMW-PAHs may damage the digestive, respiratory, and immune systems through various mechanisms [43].

## 3. Mechanisms of Intestinal Injury Induced by LMW-PAHs

The intestinal injury induced by LMW-PAHs is primarily associated with alterations in the GM, intestinal inflammation, and impairment of the intestinal barrier. Among these factors, the AhR plays a central role, and the three processes are tightly interconnected.

AhR receptors are present in the cytoplasm of intestinal epithelial cells [44] as well as various immune cells, including T cells, B cells, macrophages, dendritic cells, and innate lymphoid cells (ILCs) [45]. This ligand-activated transcription factor, AhR, exhibits prominent dual characteristics of ligand specificity and dose-dependence in its biological effects. Distinct ligands may elicit different biological effects, such that AhR activation can yield either protective or pathogenic outcomes. For instance, ligand-dependent AhR activation by 2,3,7,8-tetrachlorodibenzo-p-dioxin (TCDD) induces protective Treg differentiation and alleviates autoimmune injury, whereas 6-formylindolo [3,2-b]carbazole (FICZ) as another AhR ligand promotes pathogenic Th17 polarization and aggravates inflammatory pathology [46]. Furthermore, physiological or low-level AhR activation supports xenobiotic detoxification [47] and intestinal mucosal homeostasis, contributing to the maintenance of intestinal epithelial barrier function [48]; by contrast, high-dose or sustained AhR activation triggered by environmental toxicants drives metabolic enzyme over-induction, excessive reactive oxygen intermediate generation, disruption of the intestinal epithelial barrier, and aggravated intestinal inflammatory responses [48]. They function as key signaling mediators in the mechanism by which LMW-PAHs exert intestinal toxicity. In their inactive state, AhRs form a complex with molecular chaperones such as heat shock protein 90 (HSP90), hepatitis B virus X-associated protein 2 (XAP2), and co-chaperone p23 [49]. Studies have demonstrated that PAHs are one of the primary ligands of AhR and can activate this transcription factor [50]. Because PAHs are lipophilic toxic molecules composed of multiple benzene rings, they can enter the cytoplasm via passive diffusion and act as AhR ligands. Upon binding with PAHs, AhR undergoes conformational changes, translocates into the nucleus, and interacts with the aryl hydrocarbon receptor nuclear translocator (ARNT), subsequently regulating gene expression.

Tryptophan, an essential dietary amino acid, is metabolized by gut microbiota into derivatives such as indole, indole-3-propionic acid (IPA), and indoleacetic acid (IA) [51]. These microbial indole derivatives are identified as ligands of AhR. Under normal conditions, their activation of AhR suppresses the formation of the NLRP3 (NOD-, LRR-, and pyrin domain-containing protein 3) inflammasome, thereby inhibiting inflammation and even improving cognitive function [52]. However, since different ligands produce distinct biological responses, the binding of LMW-PAHs to AhR may upregulate cytochrome P450 1A1/1A2 expression and cause DNA damage [53], thereby altering host–microbiota metabolic functions [54]. Moreover, research indicates that AhR helps maintain intestinal epithelial barrier integrity through intracellular zinc signaling, while the presence of PAHs can disrupt these signaling pathways [55], weakening the barrier’s protective functions. Another study demonstrated that AhR signaling can promote the differentiation of intestinal stem cells into epithelial cells—an essential process for maintaining the physical and chemical barrier that defends against pathogens. Overexposure to PAHs may also activate AhR in immune cells, suppressing physiological AhR activation by normal ligands and promoting the production of pro-inflammatory cytokines. Simultaneously, it may inhibit AhR activation within Treg cells, decreasing the release of IL-10, transforming growth factor-β (TGF-β), and interleukin-4 (IL-4), thereby driving inflammatory responses [56].

From the perspective of oxidative stress, cytochrome P450 1A2 induced by LMW-PAHs–AhR binding may enhance oxidative metabolic reactions and increase reactive oxygen species (ROS) levels in intestinal epithelial cells [57] through pharmacokinetic mechanisms. Oxidative stress, in turn, can elevate intracellular calcium levels and activate upstream inflammatory signaling pathways such as NF-κB and NLRP3 inflammasomes, which stimulate the expression of pro-inflammatory cytokines [58]. These processes collectively disrupt intercellular tight junction proteins—such as occludin and zonula occludens-1 (ZO-1)—thereby damaging the intestinal barrier and increasing intestinal permeability [59].

Studies have also shown that SCFAs and other related microbial metabolites exert protective effects on the intestine through AhR, inhibiting intestinal inflammation and alleviating GM dysbiosis. Microbiota-derived SCFAs activate G-protein-coupled receptor 41 to promote interleukin-22 (IL-22) production from CD4^+^ T cells and ILCs while inhibiting histone deacetylases. SCFAs also upregulate IL-22 expression by enhancing AhR and hypoxia-inducible factor-1α signaling, thereby protecting the intestinal mucosa from inflammatory injury [60]. In contrast, exposure to LMW-PAHs disrupts these pathways by inducing GM dysbiosis and possibly suppressing SCFA-producing probiotics [61]. For instance, LMW-PAHs inhibit butyrate-producing species such as *Faecalibacterium* and *Roseburia*, leading to decreased SCFA production [62]. The reduction in SCFA levels weakens systemic immune regulation and ultimately contributes to immune disorders. In conclusion, under physiological conditions, tryptophan-derived metabolites and SCFAs activate AhR to promote IL-22 production and suppress NLRP3 inflammasome activation, thereby maintaining intestinal homeostasis, whereas upon exposure, LMW-PAHs bind to AhR and upregulate cytochrome P450 1A2, which triggers excessive ROS generation and subsequent NLRP3 activation, culminating in intestinal inflammation and barrier disruption (Figure 2).

Once the AhR is activated by LMW-PAHs, the resulting GM dysbiosis, intestinal inflammation, and barrier dysfunction interact with one another, thereby exacerbating intestinal injury. GM dysbiosis can disrupt both the antimicrobial components of the mucus layer and the tight junction proteins of the mechanical barrier [63]. Studies have indicated that GM dysbiosis and intestinal inflammation lead to Paneth cell dysfunction, resulting in reduced secretion of α-defensins and consequently increased intestinal permeability [64]. Additionally, GM dysbiosis can promote colonization by pathogenic *Escherichia coli* strains—including *E. coli* O157:H7, which produces Shiga toxin (STx) and causes hemorrhagic diarrhea—leading to reduced expression of epithelial tight junction proteins and subsequent barrier disruption [65]. Inflammatory cytokines can also impair the integrity of the intestinal barrier. For example, studies show that tumor necrosis factor-α (TNF-α) has been shown to markedly increase the expression of microRNA-122a (miR-122a) in intestinal cells. Overexpressed miR-122a binds to a motif within the 3′-untranslated region of the occludin messenger RNA (mRNA), promoting its degradation; depletion of occludin in enterocytes then increases intestinal permeability [66]. Intestinal inflammation may act both as a cause and a consequence of GM dysbiosis. Some evidence demonstrates that GM dysbiosis commonly accompanies inflammatory bowel disease (IBD) [67].

Once LMW-PAHs have caused intestinal injury, their impact is not confined to the local intestinal environment but may elicit systemic effects, contributing to injury in distal organs such as the lungs.

The core mechanism by which polycyclic aromatic hydrocarbons induce intestinal oxidative stress and inflammatory injury mediated by AhR has received relatively consistent experimental support in cell, organoid and animal models [29,44,57,59], whereas direct human evidence remains limited, and related human intestinal disease reviews also highlight this gap [63,68]. Key gaps include causal validation using germ-free animals, FMT, single-strain colonization, metabolite supplementation, and conditional AhR knockout [8,69]; meanwhile, compared to HMW-PAHs, there is still a scarcity of mechanism evidence specifically targeting LMW-PAHs. Major gut microbial metabolites that regulate gut–lung crosstalk exert dual protective and detrimental effects on intestinal and pulmonary inflammation through distinct signaling pathways (Table 2).

## 4. Mechanisms of Pulmonary Injury Induced by LMW-PAHs via the “Gut–Lung Axis”

### 4.1. Cross-Organ Communication Mechanism

Traditionally, the intestine and lungs were considered independent organs. However, recent studies have overturned this view. Because the gut and lungs share similarities in embryonic origin, anatomical structure, and immune defense mechanisms [75], and studies show that both organs possess a common mucosal immune system, they are capable of communication through highly regulated pathways [6]. Together, these interactions form a bidirectional cross-organ communication network known as the “gut–lung axis”.

Under normal physiological conditions, the intestinal barrier maintains separation between the two organs, and their interaction mainly occurs through mucosal immunity. Secretory immunoglobulin A (SIgA) produced at inductive sites can migrate and home to different mucosal effector sites throughout the body, enabling immune responses against common antigens [6]. In this way, SIgA neutralizes pathogens and prevents their adhesion to epithelial surfaces [76]. During intestinal inflammation, excessive SIgA secretion may be stimulated to counteract pathogens; however, the specificity of SIgA may decline, hindering efficient pathogen clearance [74].

Beyond mucosal immunity, other communication pathways exist between the gut and lungs. GM ferment dietary fiber to produce SCFAs, including acetate, butyrate, and propionate. On one hand, the lipid-soluble nature of SCFAs allows passive diffusion across cells into the portal circulation; on the other hand, intestinal epithelial cells express G-protein-coupled receptors GPR41, GPR43, and GPR109A that mediate SCFA absorption into the bloodstream [77]. When SCFAs are transported to the lungs, they enhance immune cell function—such as the phagocytic and bactericidal activity of alveolar macrophages—and help attenuate pulmonary inflammation [78]. However, the activation of the AhR by LMW-PAHs results in dysbiosis of the GM, subsequently diminishing SCFA production. This disruption weakens immune regulation along the gut-lung axis, thereby increasing susceptibility to lung inflammation. Moreover, trimethylamine *N*-oxide (TMAO), a downstream metabolite of GM, is produced through the catalytic activity of TMA lyase and can further induce systemic inflammatory responses. When LMW-PAHs activate AhR and disrupt gut microbiota balance, intestinal TMAO production may increase [79]. Research indicates that intestinal barrier impairment is a prerequisite for the migration of intestinal immune cells to extraintestinal sites [80]. Activation of AhR by LMW-PAHs that compromises the intestinal barrier causes aberrant homing of intestinal immune cells—including α4β7^+^, CCR9^+^, and CD4^+^ T cells—to the lungs, potentially driving pulmonary inflammation [81]. In cases of sepsis, for instance, gut-derived memory γδ T17 cells have been observed to migrate to the lungs and release interleukin-17A (IL-17A), aggravating lung inflammation [82].

Furthermore, gut inflammation triggered by LMW-PAHs can lead to the release of multiple metabolites (such as TMAO) and inflammatory mediators—including LPS, TNF-α, interleukin-6 (IL-6), interleukin-1β (IL-1β) [83], IL-10, and interferon-γ (IFN-γ) [84]—which can circulate through the blood stream and reach distal organs such as the lungs, causing various levels of injury.

Because the communication network is bidirectional, it not only includes pathways through which gut signals influence the lungs but also feedback mechanisms whereby pulmonary inflammation affects the gut. Studies have shown that lung infection or inflammation leads to the release of inflammatory cytokines such as TNF-α, IL-6, and IL-1β [85]. These mediators can diffuse through the bloodstream, influencing distant organs, including the intestine. Pulmonary inflammation can also affect the number and function of intestinal immune cells, thereby altering gut immune homeostasis and causing secondary intestinal injury [85]. In summary, immune cells and cytokines generated in the intestinal tract can enter lymphatic and blood circulation, travel via the heart and pulmonary artery, and finally reach the alveoli to exert immunological effects, which illustrates the gut-lung axis-mediated cross-organ communication (Figure 3).

The vagus nerve constitutes an essential bidirectional communication pathway connecting the gut and the central nervous system (CNS) [86]. It links the gastrointestinal tract with the CNS, transmitting signals derived from the intestine to the brain and, conversely, modulating gastrointestinal function [87]. Afferent vagal fibers are responsible for sensing physical and chemical stimuli from the gastrointestinal tract, including nutrients, microbial metabolites such as SCFAs, and gut hormones. These signals are then transmitted via the vagus nerve to the nucleus tractus solitarius (NTS) and other brainstem nuclei [88]. Efferent vagal fibers regulate multiple aspects of pulmonary and systemic physiology, including airway tone, blood flow, mucus secretion, and respiratory patterns. They also release neuropeptides that modulate immune cell activity and control lung inflammation through the cholinergic anti-inflammatory pathway [89].

In addition, enterochromaffin cells (ECCs) play pivotal roles in neuroendocrine signaling within the gut. ECCs can sense gut contents and secrete 5-hydroxytryptamine (5-HT, serotonin) into the bloodstream [90]. 5-HT promotes inflammatory responses by enhancing ROS generation via NADPH oxidase 2 and by increasing the secretion of cytokines such as IL-6 and interleukin-8 (IL-8). It also stimulates the production of monocyte chemoattractant protein-1 (MCP-1), which facilitates adhesion of inflammatory monocytes to colonic epithelial cells, ultimately contributing to widespread intestinal inflammation [91].

Current evidence is largely derived from animal models and indirect human observations [81,85,92], and the quantitative and temporal contributions of individual mediators remain unclear [92]. Key gaps include determining the relative contribution of each mediator and validating these pathways in other exposure models and population cohorts of LMW-PAHs [8,81]. Major functional mediators participating in bidirectional gut–lung crosstalk, including microbial metabolites, immune cells, inflammatory factors, and neurotransmitters, are summarized below (Table 3).

### 4.2. Mechanisms of Pulmonary Injury

As the organ responsible for gas exchange, the lungs possess an extensive network of capillaries. The endothelium of these pulmonary capillaries is composed of a single, extremely thin layer of cells—approximately 0.2 µm in thickness [97]. While this structure facilitates efficient gas exchange, it also renders the lung more susceptible to damage by circulating inflammatory mediators. Moreover, because of the structural and functional similarities between the intestinal and airway mucosa, both tissues express homologous receptors such as chemokine ligand 28 (CCL28) and chemokine receptor 9 (CCR9) [92]. These receptors allow lymphocytes to migrate bidirectionally between the intestine and airways via the bloodstream, thereby potentially contributing to pulmonary inflammation.

Once inflammatory mediators reach the lungs through blood circulation, they can activate immune cells and inflammatory signaling pathways, thereby initiating pulmonary inflammation. For instance, LPS activates the Toll-like receptor 4 (TLR4)/myeloid differentiation factor 88 (MyD88)/interleukin-1 receptor-associated kinase (IRAK)/tumor necrosis factor receptor-associated factor 6 (TRAF6)/NF-κB signaling cascade in bronchial epithelial cells, leading to the production of pro-inflammatory cytokines such as IL-6, TNF-α, and IL-1β. This process may trigger acute lung injury (ALI) [98]. Concurrently, LPS can increase epithelial expression of Fas and Fas ligand (FasL), inducing apoptosis and compromising the integrity and barrier function of the pulmonary epithelium [99].

In addition, the gut microbial metabolite TMAO may activate macrophages and monocytes, stimulating the release of chemokines and cytokines that further drive pulmonary inflammation. Persistent pulmonary inflammation has been associated with the development or progression of respiratory diseases such as COPD [100], cystic fibrosis [101], pneumonia [102], asthma [102], lung cancer [103], acute lung injury, and acute respiratory distress syndrome (ARDS) [104], as well as with systemic diseases.

Current mechanistic evidence is mainly from animal and cell models [79,98,99] whereas human evidence is largely associative with respiratory diseases such as COPD and ARDS [100,102,104]. Key gaps include establishing a direct oral model of lung injury caused by LMW-PAHs and verifying the causal role of the relevant pathways in the pathogenic process.

Inflammatory condition that can progress to multiple organ dysfunction syndrome (MODS), shock, and even death [105].

Pulmonary inflammation may also send feedback signals to the central nervous system through neuroendocrine communication, influencing the intestine [106].

In-depth investigation of how LMW-PAHs affect the composition and function of GM and how GM-derived metabolites mediate pulmonary injury through the gut–lung axis will be crucial for developing novel preventive and therapeutic strategies. To conclude, LMW-PAH exposure induces gut microbiota dysbiosis, oxidative stress and intestinal barrier damage; inflammatory mediators and LPS then enter the circulation, activate the TLR4/NF-κB pathway in bronchial epithelial cells and trigger systemic inflammation, leading to multi-organ injuries including enteritis and pneumonia (Figure 4). Key pathological mechanisms, phenotypic manifestations, and adverse health outcomes associated with LMW-PAH-induced gut–lung axis disruption are summarized below (Table 4).

## 5. Mechanisms of Probiotic Intervention

Given that the pathogenic effects of LMW-PAHs are mainly mediated through local intestinal inflammation and dysbiosis of the GM, leading to intestinal barrier impairment, systemic dissemination of inflammatory cytokines, and ultimately pulmonary inflammation, probiotics such as *Lactobacillus acidophilus*, *Bifidobacterium breve*, and *Saccharomyces boulardii* have been shown to possess anti-inflammatory, gut microbiota-regulating, and immunomodulatory properties [111]. These mechanisms may partially counteract the pathogenic processes induced by LMW-PAHs. Although direct studies on the modulation of LMW-PAH-induced gut–lung axis toxicity by probiotics remain limited, current evidence suggests that probiotics can regulate GM composition, enhance immune function, and attenuate inflammation, providing both a theoretical and practical foundation for mitigating LMW-PAH toxicity [112].

### 5.1. Mechanisms by Which Probiotics Regulate Gut Microbiota Composition

The primary effect of probiotic intervention is to increase beneficial bacteria while reducing harmful bacteria [113]. Probiotics exert these effects mainly through competitive exclusion and production of antimicrobial substances, thereby inhibiting the proliferation of opportunistic pathogens. Moreover, probiotics facilitate the colonization and growth of beneficial bacterial strains, indirectly restoring gut microbial homeostasis [113].

The competitive exclusion mechanism of probiotics involves three main aspects: (1) competition for adhesion sites, (2) competition for nutrients, and (3) secretion of antimicrobial compounds. Firstly, mucins, secreted by some epithelial cells such as goblet cells, are high-molecular-weight glycoproteins that form adhesion substrates within the intestinal mucus layer. In the small and large intestines, mucins are primarily composed of MUC2 [114]. These mucins serve as attachment sites for GM. Upon probiotic administration, a higher proportion of beneficial bacteria compete with harmful bacteria for these binding sites, thereby inhibiting pathogenic adhesion [115]. Furthermore, studies indicate that GM may influence mucin production; for example, *Streptococcus mutans* and *S. sobrinus* stimulate mucin secretion of intestinal epithelial cells, whereas *Lactobacillus reuteri* suppresses it [116]. In addition, bacteria synthesize adhesins to mediate colonization in the intestinal environment. Boris Dudík et al. demonstrated that *Lactobacillus reuteri* expresses specific surface adhesins such as mucus-binding protein (MUB) and mucus adhesion-promoting protein (MapA), enhancing its mucosal adherence and competitively excluding pathogens [117]. Secondly, the GM can recover energy through fermentation of undigested food residues [118]. When probiotics are introduced, beneficial bacteria compete more effectively for these nutrient sources, inhibiting the growth of pathogenic species. *Bifidobacterium* is known to secrete extracellular glycosidases, efficiently degrading carbohydrates and thereby gaining a metabolic advantage over pathogens in substrate competition [119]. Thirdly, antibiotic substances such as organic acids and bacteriocins play critical roles in the GM. Lactic acid—an important metabolite of *Lactobacillus*—lowers intestinal pH and inhibits the growth of acid-sensitive pathogens [120]. Studies have shown that *Lactobacillus* strains CKCC1858 and CKCC1312 may increase SCFAs by modulating SCFA-producing microbial abundance, further decreasing intestinal pH and inhibiting the growth of pathogenic organisms that exhibit weak acid resistance. [121]. Research indicates that nearly all bacteria produce bacteriocins as part of innate defense mechanisms. As antimicrobial peptides, bacteriocins exhibit antibiotic-like activity by altering enzyme activity, inhibiting spore germination, deactivating anionic carriers, or forming pores in cell membranes [122]. Therefore, when beneficial bacteria dominate the GM, bacteriocin production provides additional protection against pathogen invasion.

Through the modulation of GM structure—reducing pathogen invasion and increasing beneficial bacterial abundance—probiotics can indirectly strengthen the intestinal barrier and enhance mucosal immune function.

### 5.2. Molecular Mechanisms by Which Probiotics Enhance Intestinal Barrier Function

Probiotics can reinforce the intestinal barrier by enhancing epithelial cell function and conducting immune signaling, thereby preventing intestinal leakage and alleviating damage caused by LMW-PAHs [123].

Probiotics ferment undigested polysaccharides to produce SCFAs such as acetate, propionate, and butyrate [124]. Studies have shown that, in addition to *Lactobacillus reuteri* [125], *Akkermansia muciniphila* can also generate SCFAs—particularly butyrate and isovaleric acid [126]—while *Bifidobacterium* species are also major SCFA producers [61]. SCFAs serve as the preferred energy substrates for colonic epithelial cells [127]. Isotope-tracing studies have demonstrated that SCFAs are rapidly absorbed in the proximal colon and converted into glucose and acetyl-CoA to nourish colon epithelial cells [128]. Colon epithelial cells can produce tight junction proteins such as occludin, claudin, junctional adhesion molecules (JAMs), and tricellulin to reinforce the intestinal physical barrier [129]. Apart from this physical defense with the strongest isolation function, specialized colonic epithelial cells, including Paneth and goblet cells, secrete chemical mediators that strengthen the chemical barrier. The mucus layer, as the most important component of the chemical barrier, forms a sieve-like gel structure, which can prevent pathogens from directly contacting intestinal epithelial cells [130]. Among them, goblet cells produce gel-like mucus, which is mainly composed of mucin (MUC2) and can isolate pathogenic bacteria [131]. Paneth cells can secrete antimicrobial peptides such as defensins. Defensins are peptide substances with broad-spectrum antibacterial activity and can directly kill or inhibit pathogenic microorganisms [132]. Moreover, microfolded cells can transport large lumen antigens and microbiota to the lamina propria of the intestine, allowing the immune cells in the lamina propria to function [133].

In addition, when pathogens penetrate the intestinal lamina propria, they activate immune cells to release cytokines such as IL-22, which bind to IL-22 receptors on epithelial cells, upregulating tight junction protein expression (e.g., claudin-2). Both in vitro and in vivo studies have confirmed that IL-1, IL-6, IL-13, and TNF may enhance claudin-2 expression [134].

### 5.3. Roles of Probiotics in Immune Homeostasis and Anti-Inflammatory Regulation

In other models, probiotics have been reported to increase local intestinal immunoglobulin A (IgA) levels through SCFA production [135]. IgA plays a pivotal role in mucosal immunity by neutralizing pathogens and toxins [113], thereby preventing infection. Furthermore, SCFAs can modulate macrophage function and mitigate inflammation. Experimental studies by J. M. Li et al. demonstrated that butyrate-producing probiotics induce macrophage polarization toward the anti-inflammatory M2 phenotype, thereby suppressing the inflammatory response [136,137].

Importantly, SCFAs may also act through the AhR to decrease the Th17/Treg ratio, reducing pro-inflammatory cytokine production. For example, *Lactobacillus murinus* modulates host tryptophan-derived indole metabolites and SCFA levels, activating the AhR pathway and inhibiting HDAC activity, which upregulates the expression of forkhead box protein P3 (Foxp3) [69]. Increased Foxp3 promotes differentiation of CD4^+^ T cells into Treg cells, which in turn suppress conversion into Th17 cells [138]. Th17 cells secrete pro-inflammatory cytokines such as IL-17, IL-21, and IL-22, whereas Treg cells secrete anti-inflammatory cytokines such as IL-10 and TGF-β [139,140]. Activated macrophages, T cells, and natural killer cells further produce TNF-α, often stimulated by Th17 activation [141].

In addition to SCFA–AhR signaling, studies indicate that fecal microbiota transplantation (FMT) restores GM composition and activates the Janus kinase/signal transducer and activator of transcription (JAK/STAT) pathway, thereby promoting Foxp3 transcription, improving Th17/Treg balance, and alleviating LPS-induced intestinal and pulmonary injury in animal models [142]. Conversely, exposure to LMW-PAHs may raise the Th17/Treg ratio, leading to immune imbalance and exacerbated inflammation [143].

Oxidative stress is a key factor in many inflammatory diseases, and the antioxidant activity of probiotics helps protect cells and tissues from LMW-PAH-induced damage. *Lactobacillus* species can suppress ROS generation, thereby mitigating oxidative stress [144]. Lactic acid bacteria (LAB) enhance the antioxidant capacity of fermented products through various biochemical processes. During fermentation, certain strains release free phenolic compounds that interrupt oxidative chain reactions. In protein-rich environments, LAB catalyze the breakdown of proteins into small peptides and free amino acids with potent antioxidant properties that neutralize ROS. These antioxidative peptides have been identified in probiotic yogurt and fermented milk, where they function by chelating pro-oxidant metal ions [145]. In contrast, pathogenic metabolites such as LPS and TMAO can disrupt the thioredoxin and glutathione systems, exacerbating cellular oxidative stress [146].

Because probiotics regulate the GM and limit pathogen invasion, they reduce activation of the NF-κB signaling pathway induced by inflammatory cytokines (e.g., TNF-α [147]) and reactive oxygen species [148], thereby dampening inflammatory amplification. Otherwise, downregulating malondialdehyde (MDA) levels and enhancing superoxide dismutase (SOD) activity further relieve oxidative stress and suppress expression of TLR, MyD88, and NF-κB, producing anti-inflammatory benefits [126]. Normally, NF-κB remains inactive when bound to IκBα; upon stimulation by TNF-α or LPS, IκBα becomes phosphorylated, ubiquitinated, and degraded, freeing NF-κB to regulate gene transcription [149,150]. NF-κB controls numerous genes associated with immunity and inflammation [151]; its sustained hyperactivation contributes to chronic inflammatory processes [152]. LMW-PAH exposure elevates pro-inflammatory cytokines (e.g., TNF-α, IL-6, IL-1β) while decreasing anti-inflammatory IL-10 [153], leading to excessive NF-κB activation. Probiotic intervention may inhibit this pathway, thereby alleviating inflammation.

Moreover, long-chain fatty acids such as palmitic acid, derived from metabolites of *Lactobacillus paracasei*, can indirectly inhibit the occurrence of inflammation. These compounds activate immune cell surface G-protein-coupled receptors (GPR40/GPR120) and downstream signaling cascades, promoting early immune defense by regulating the release of IL-1β and other mediators. Palmitic acid can also function as a Toll-like receptor (TLR) ligand to activate dendritic cells and macrophages, thus strengthening immune responsiveness [154].

Probiotics have been proposed to play a role in the enterohepatic circulation of bile acids, converting primary into secondary bile acids through the actions of bile salt hydrolase (BSH) and 7-dehydroxylase [155]. Secondary bile acids act as signaling molecules that activate the farnesoid X receptor (FXR) and Takeda G-protein-coupled receptor 5 (TGR5) on host cells [156]. FXR activation suppresses macrophage IL-6 expression [157], while TGR5 activation inhibits the NF-κB signaling pathway, thereby attenuating inflammation [158]. To conclude, probiotics remodel gut microbiota composition, promote SCFA production, enhance intestinal tight junction integrity, and modulate multiple signaling pathways, including TLR2/NF-κB, iron metabolism and ferroptosis, to exert anti-inflammatory and antioxidative effects and maintain intestinal immune homeostasis (Figure 5).

However, direct evidence for counteracting LMW-PAH-induced lung injury through the gut–lung axis is limited [69]. The key research gaps include the fact that the effects of probiotics vary among different strains, and the appropriate intervention dosage and treatment duration are not yet unified. More high-quality randomized controlled trials are still needed [159]. Key intervention mechanisms and evidence types regarding probiotic-mediated gut–lung axis regulation against LMW-PAH toxicity are summarized below (Table 5).

## 6. Summary and Perspectives

This review clarifies the cascade by which oral exposure to LMW-PAHs induces pulmonary injury through the “gut–lung axis”: LMW-PAHs disrupt gut microbiota homeostasis, compromise the intestinal barrier, and trigger local and systemic inflammation that propagates to the lungs. Among these processes, ligand-dependent AhR signaling plays a central, dual role, driving intestinal oxidative stress and inflammatory injury. As a potential countermeasure, probiotics may attenuate this toxicity by modulating gut microbiota composition, reinforcing the intestinal barrier, regulating immune homeostasis, and producing protective metabolites such as short-chain fatty acids.

However, the current evidence base remains largely mechanistic and indirect. Direct studies of probiotic intervention against LMW-PAH-induced gut–lung toxicity are still limited, and quantitative data on actual dietary LMW-PAH exposure and its relevance to experimental doses remain scarce. Key gaps therefore include establishing causality through germ-free models and fecal microbiota transplantation, defining effective strains, doses, and treatment duration, and validating efficacy in high-exposure populations through randomized controlled trials.

Addressing these gaps will require integrative approaches—metagenomics, metabolomics, and single-cell technologies—to map the underlying molecular networks. Ultimately, a multilevel strategy combining source control, reduced exposure, and validated probiotic interventions may offer a route to mitigating LMW-PAH-associated respiratory risk.

## Figures and Tables

**Figure 1:**
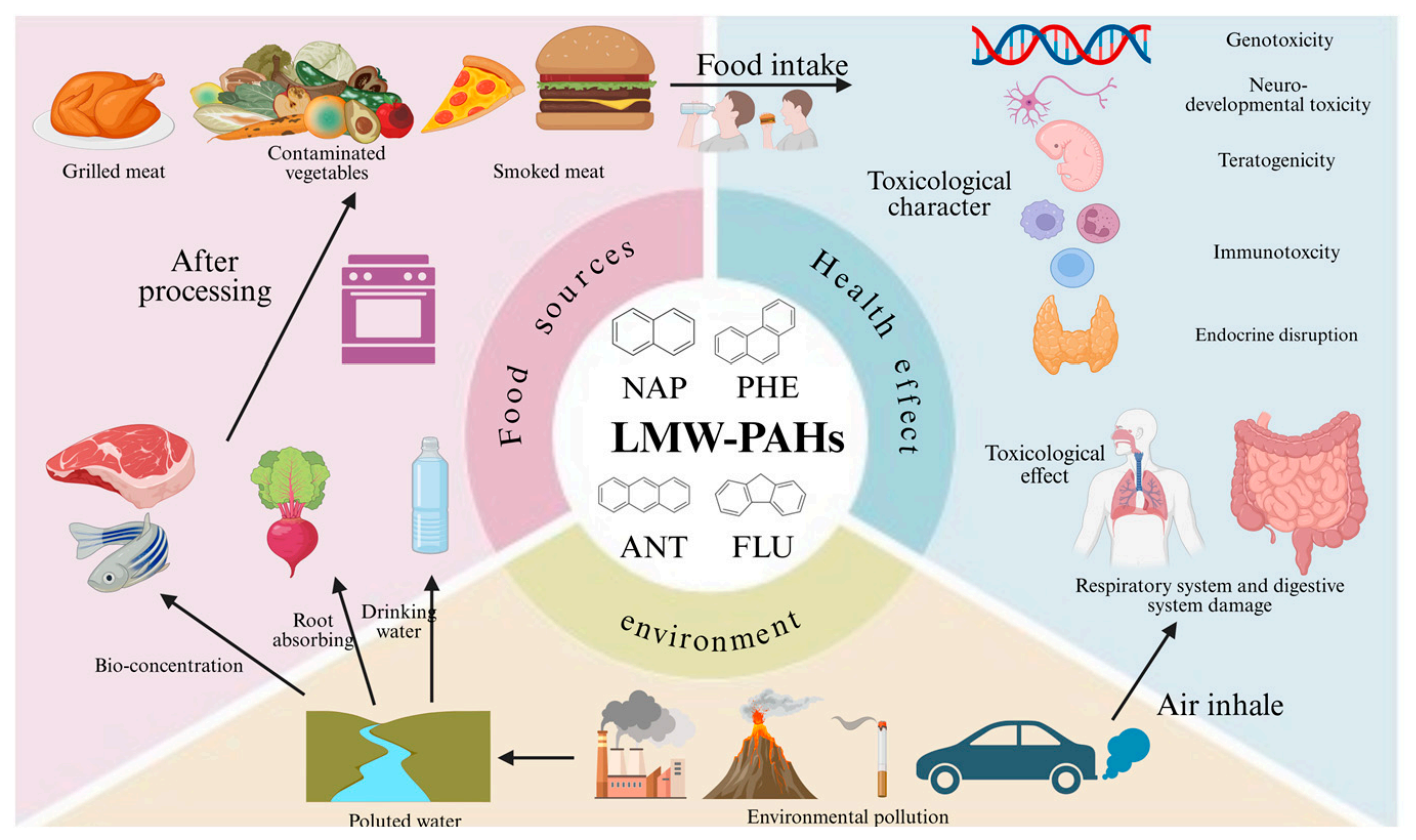
Sources and toxic effects of LMW-PAHs.

**Figure 2:**
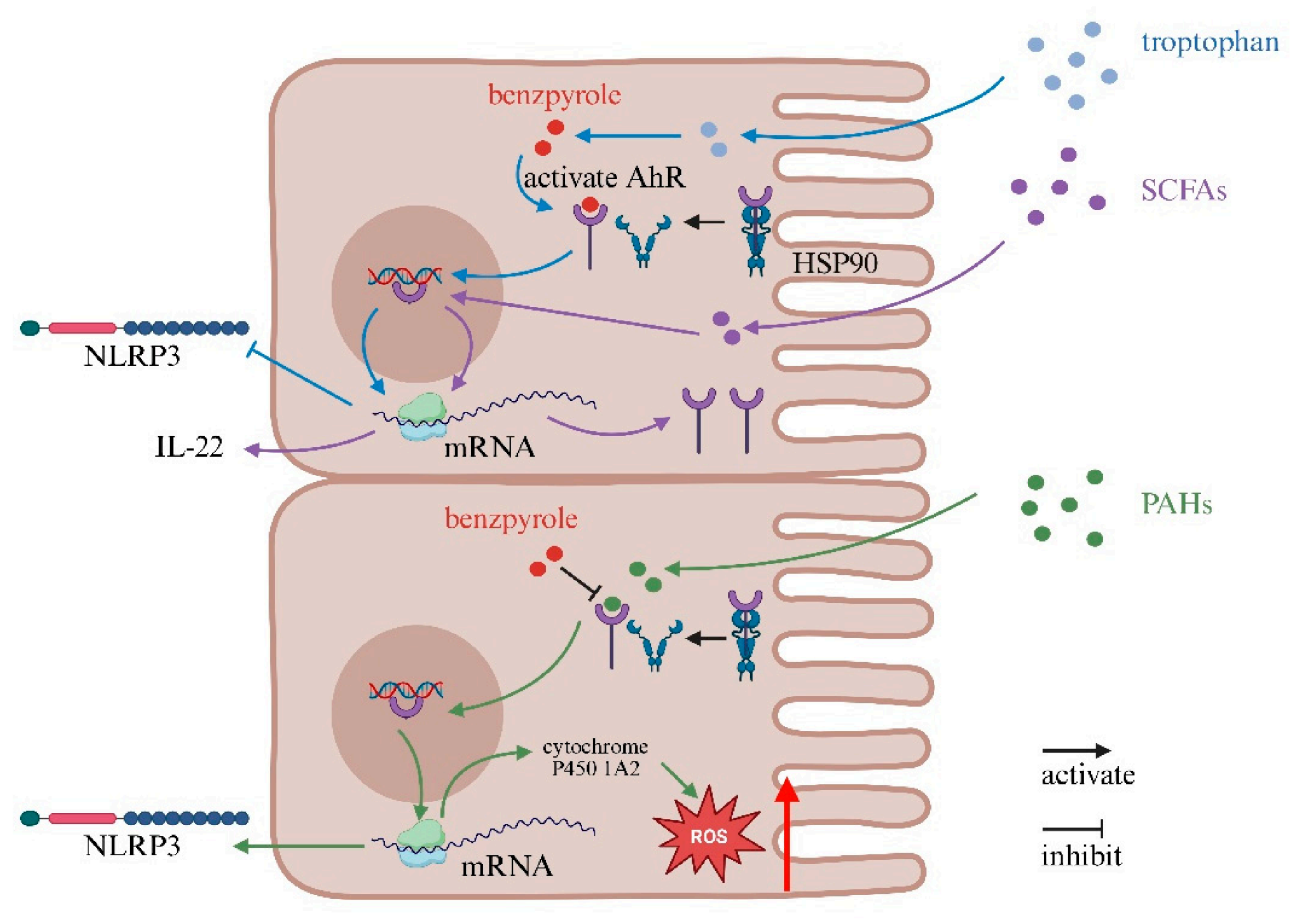
Mechanistic role of AhR in LMW-PAH-induced intestinal injury.

**Figure 3:**
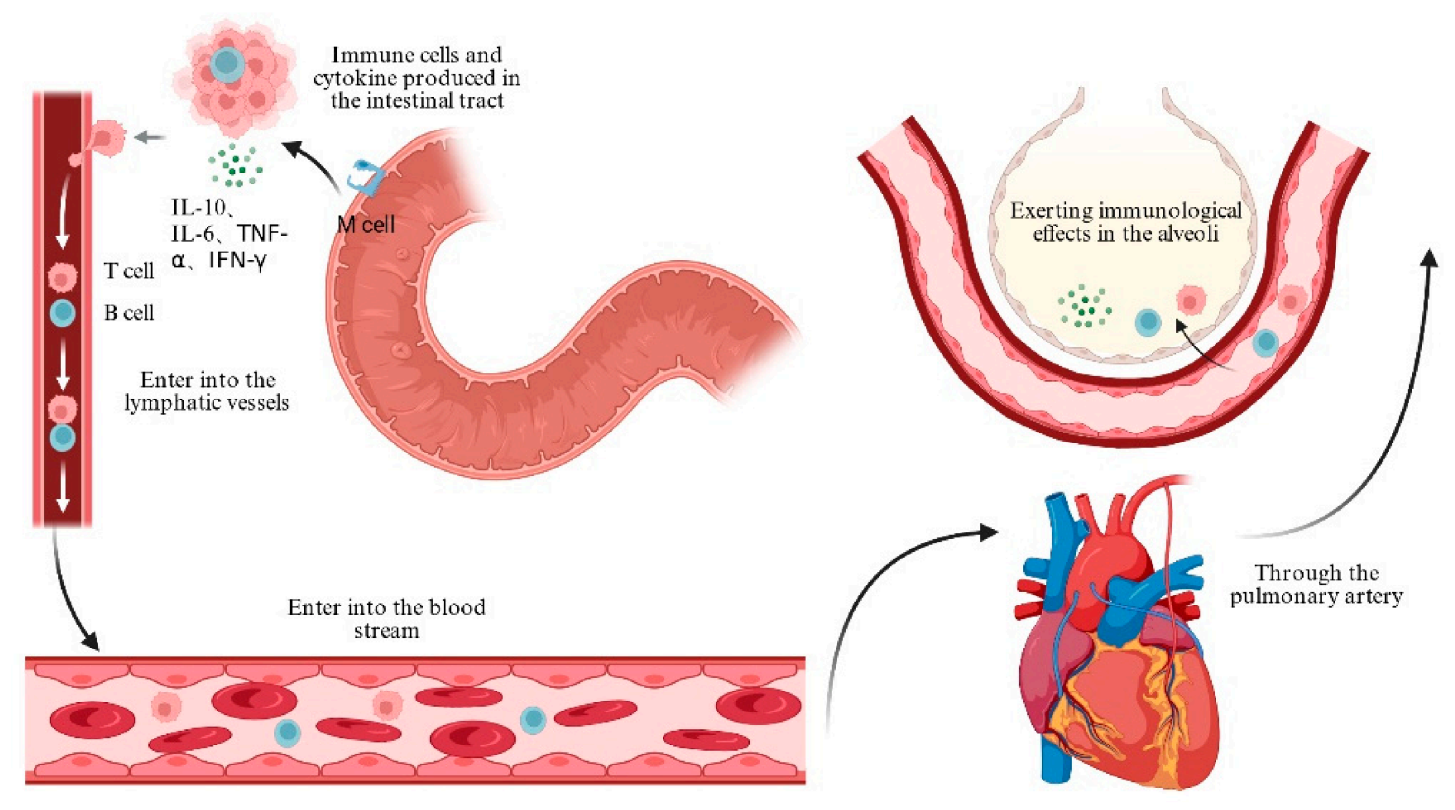
Schematic diagram of shared mucosal immunity between the gut and lung.

**Figure 4:**
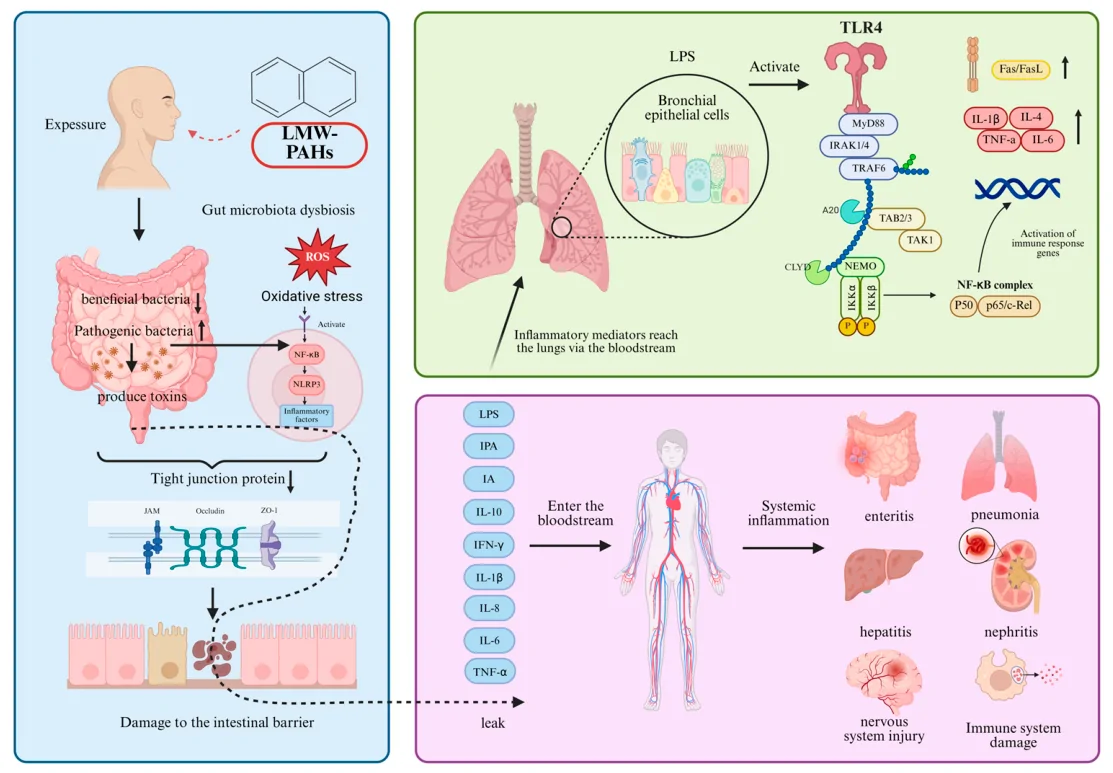
Possible mechanisms by which oral exposure to LMW-PAHs disrupts the gut–lung axis.

**Figure 5:**
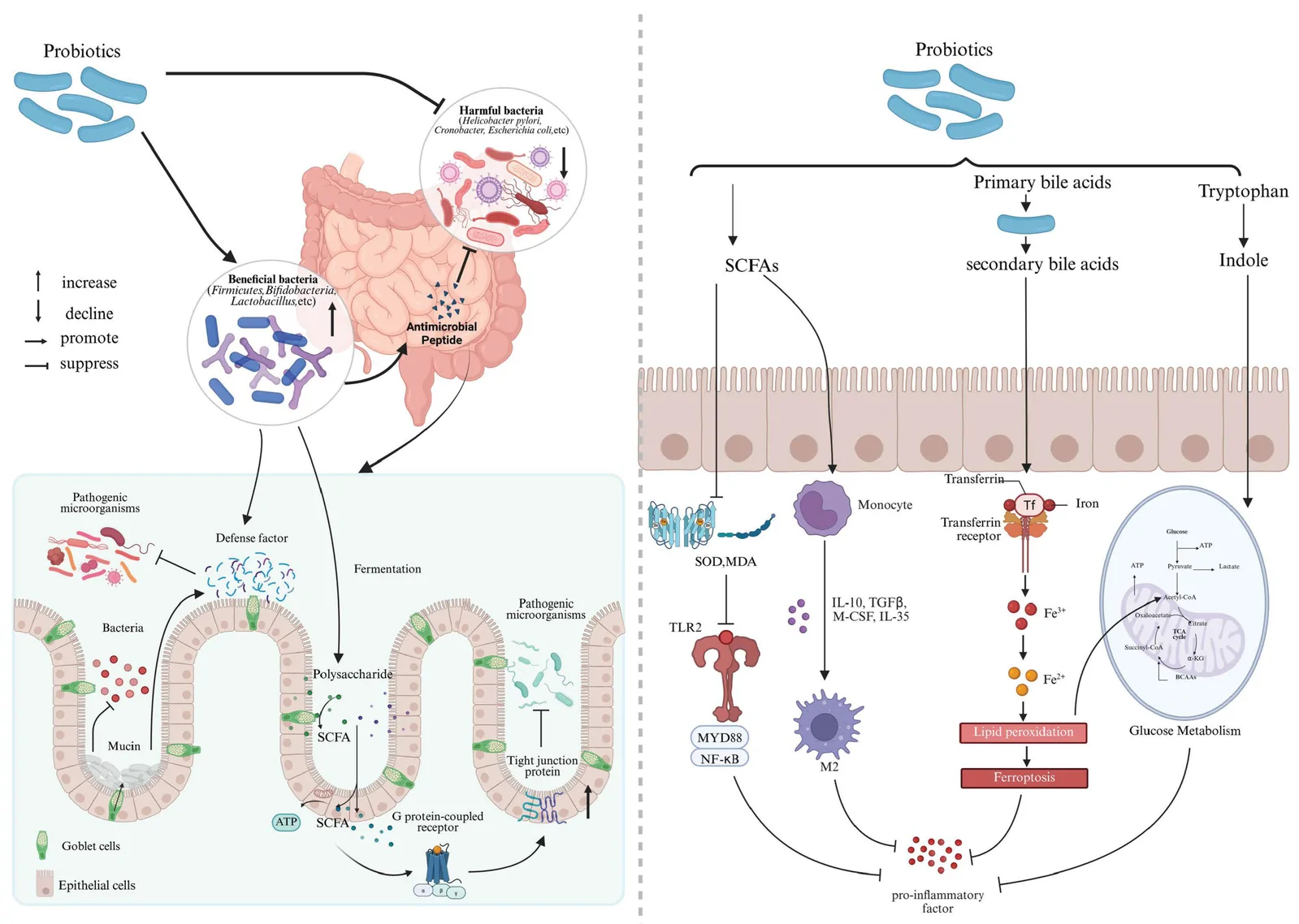
Mechanisms by which probiotic intervention attenuates gut–lung axis toxicity induced by LMW-PAHs.

**Table 1:** Common characteristics and main health impacts of LMW-PAHs.

Name	Structure	Main Source	Main Exposure Pathway	Major Hazards	Research Source
Naphthalene	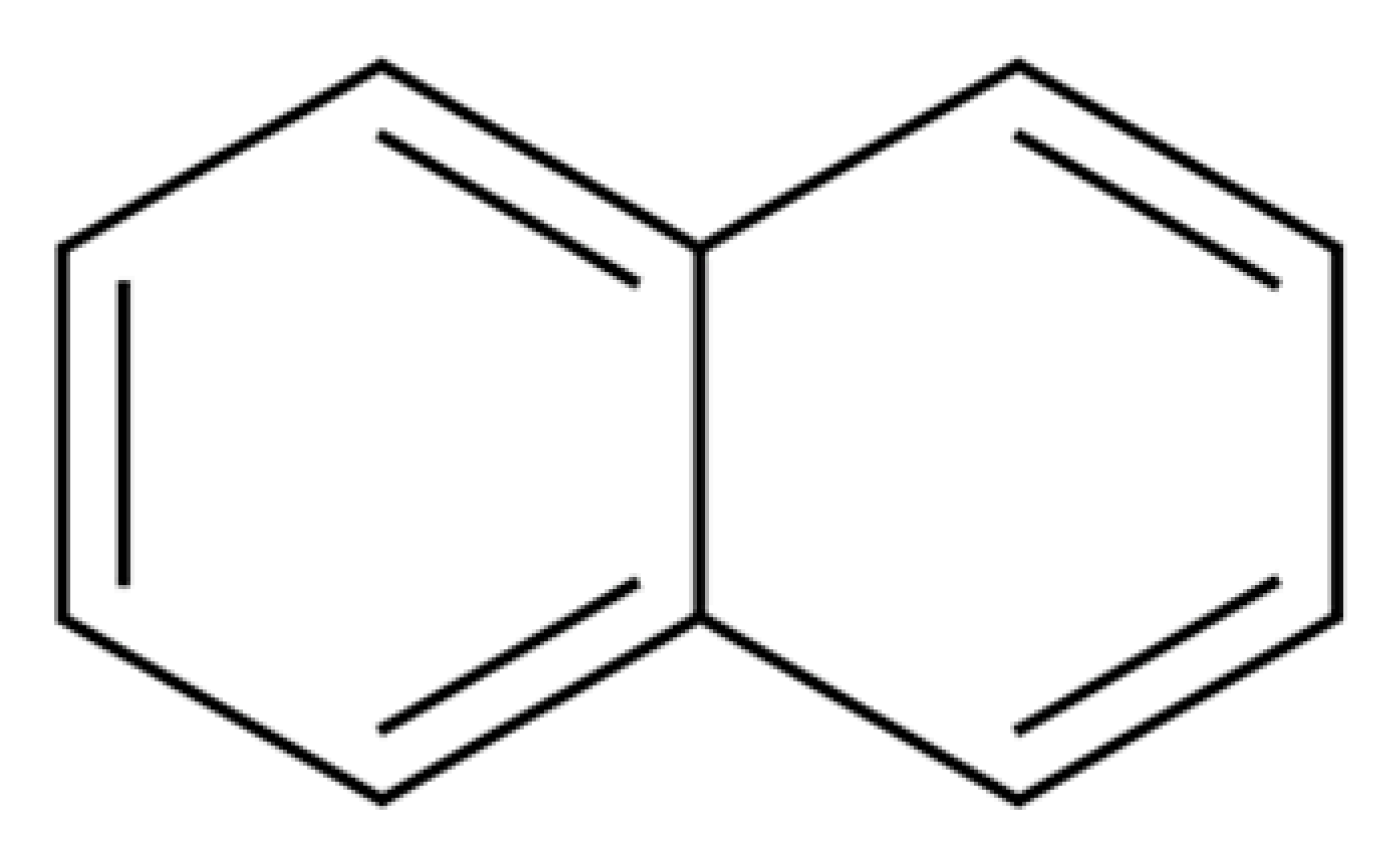	Fossil fuels (coal, oil), insecticides, and deodorants	Inhalation	Respiratory system, reproductive system, and immune system damage	[24]
Anthracene	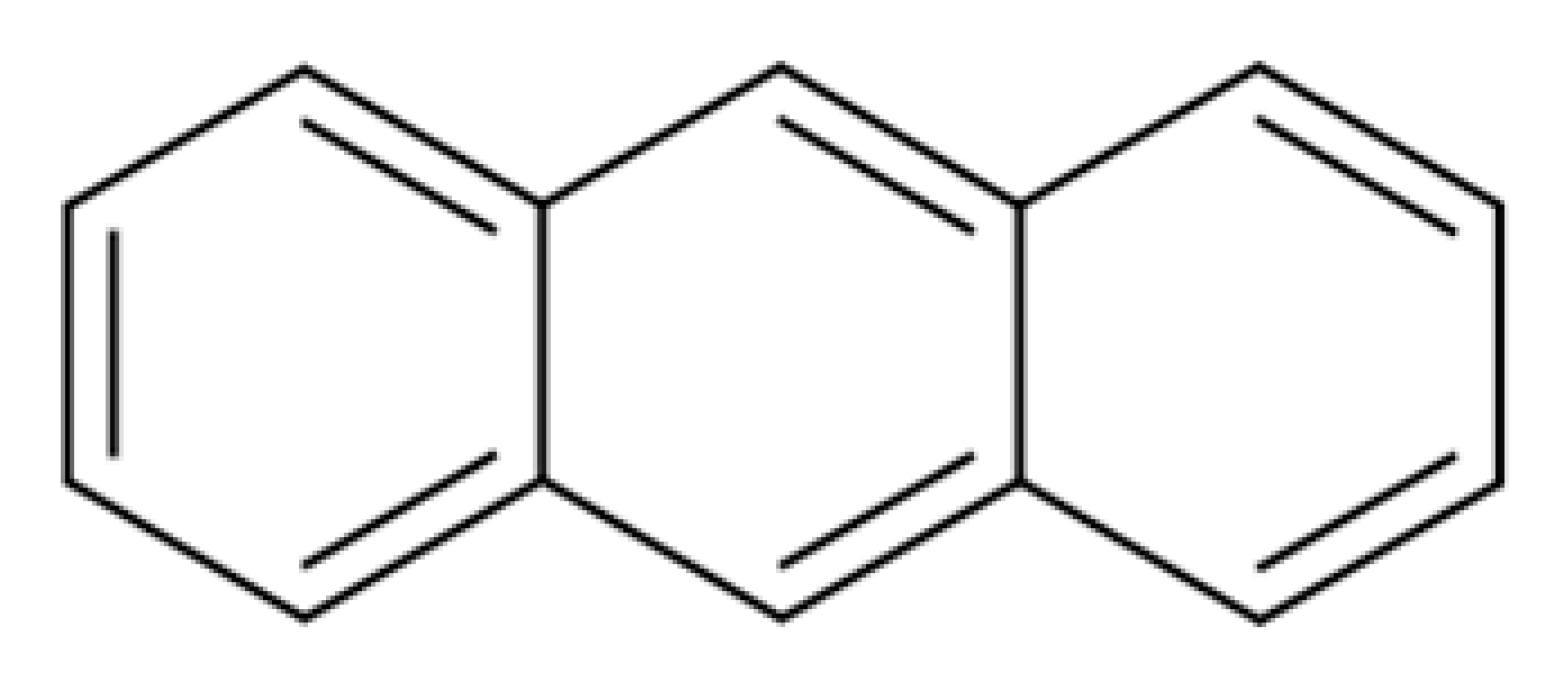	Industrial materials, pesticides, wood preservatives, and plant growth regulators	Inhalation, ingestion of contaminated food	Carcinogenicity	[25]
Phenanthrene	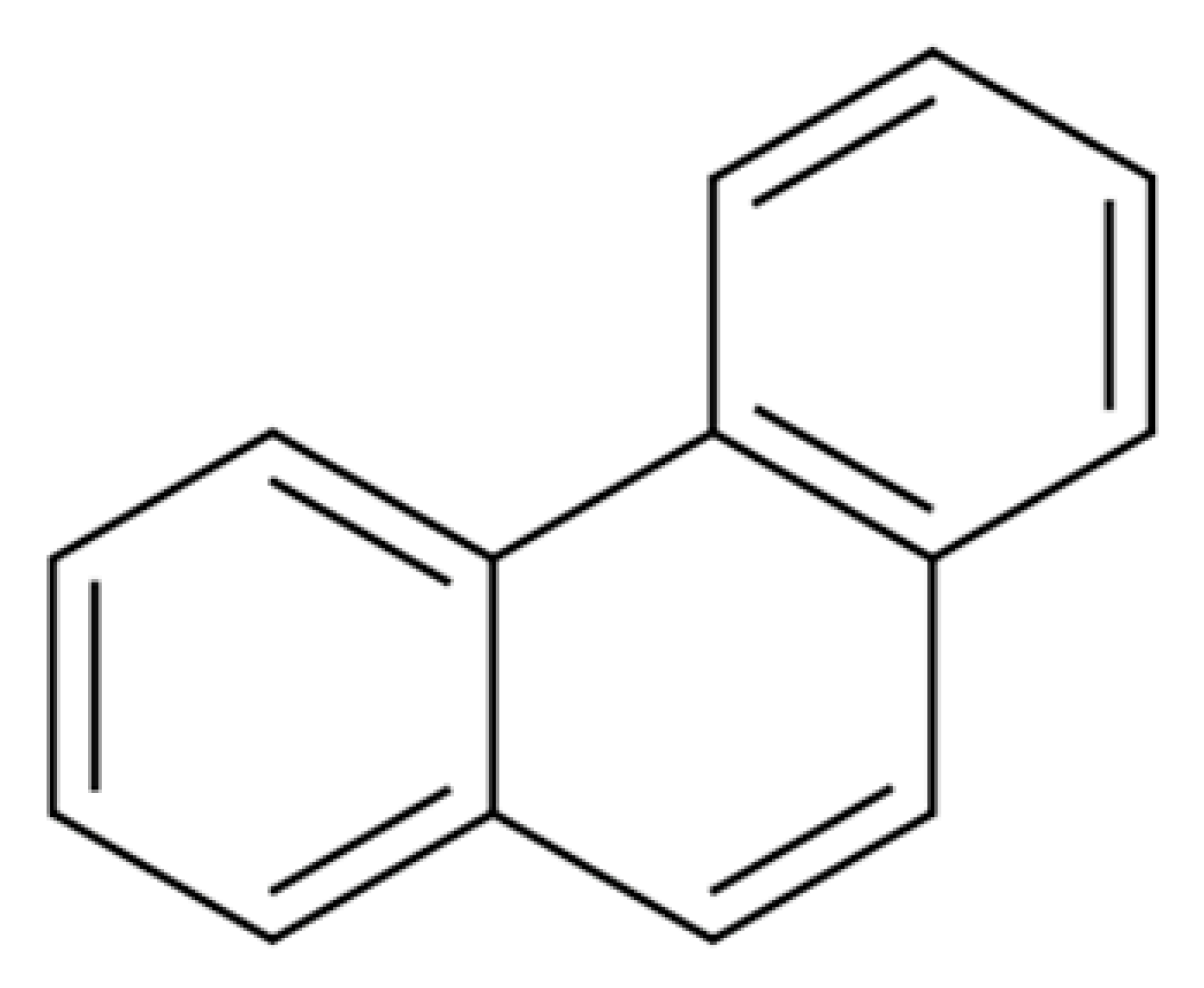	Combustion products of coal, ash	Inhalation, ingestion of contaminated food	Genetic toxicity, DNA damage	[26]
Fluorene	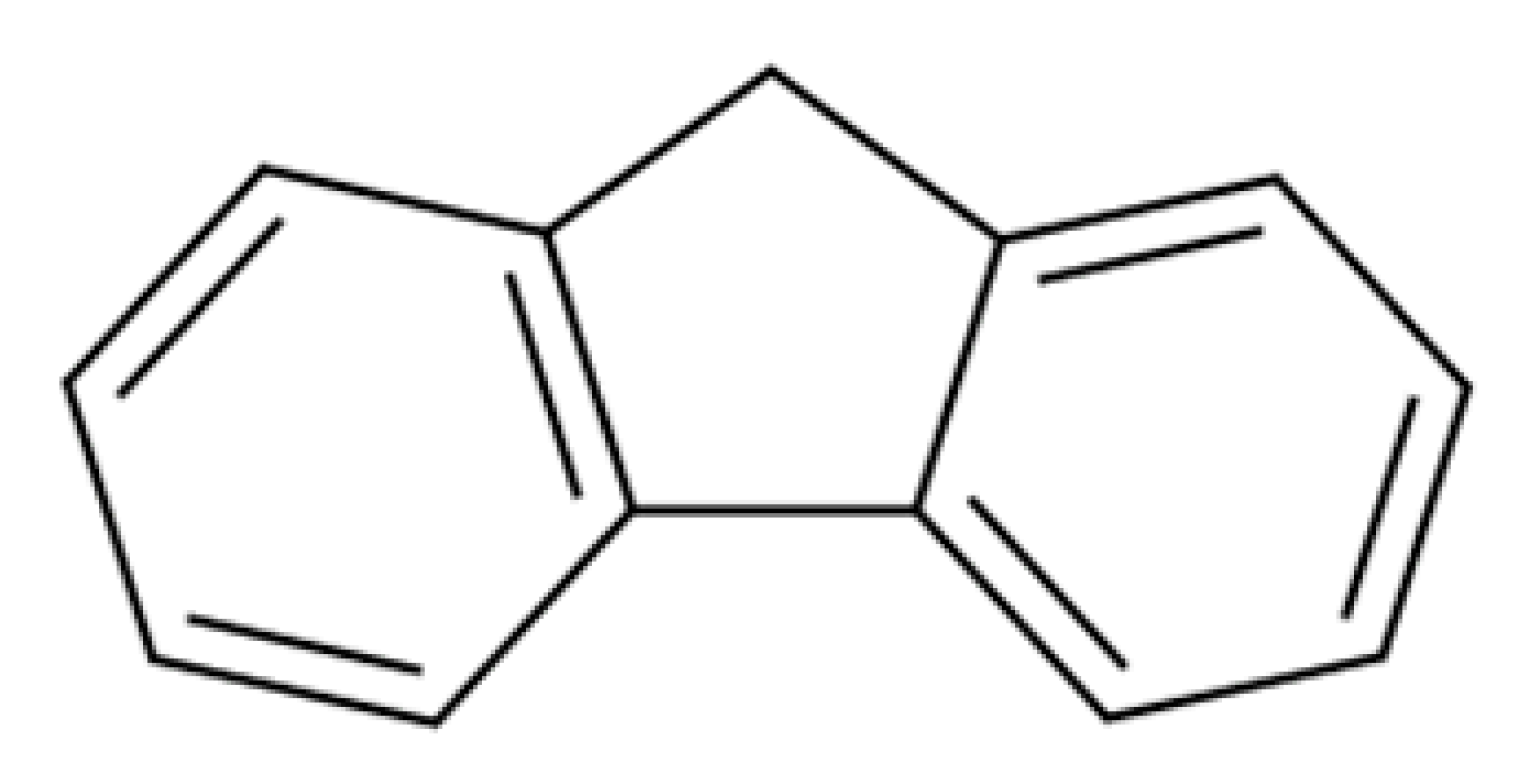	Soil and fossil fuel combustion	Ingestion of contaminated food, inhalation, and dermal contact	Neurotoxic effects, genetic toxicity, behavioral disorders	[27]

**Table 2:** Main microbial metabolites in the gut-lung axis and their functions.

Metabolites	Main Producing Bacteria	Effects on the Intestine	Effects on the Lungs	Related Signaling Pathways	Research Source
Short-chain fatty acids	*Bifidobacterium*	Beneficial to intestinal barrier integrity, alleviates intestinal inflammation	Alleviates pulmonary inflammation	Induces reduction of pro-inflammatory cytokines, inhibits histone deacetylases (HDACs)	[70]
Indoleacetic acid (IAA)	*Escherichia coli*, *Bacillus* and *Bacteroides*	Enhances intestinal immune barrier function	Attenuates pulmonary fibrosis	Activates the aryl hydrocarbon receptor (AhR), initiating the expression of genes such as IL-22, IL-17, and IL-10	[71]
Succinate	*Escherichia coli*, succinate-producing *Firmicutes* (succinate-producing lineages)	Inhibits proliferation of intestinal mucosal epithelial cells	Induces pulmonary fibrosis	Affects glycolysis and TCA cycle function via the succinate-GPR91 axis	[72]
D-lactate	*Lactobacillus murinus* and other lactic acid bacteria	Causes D-lactic acidosis, leading to shortening of intestinal villi	May induce pulmonary inflammation	Induces secretion of IL-6 and IL-8; induces phosphorylation of MAPK, ERK, and activates the NF-κB pathway	[73]

For D-lactate, the association with pulmonary inflammation is a mechanistic hypothesis inferred from studies in cattle [74] and has not been directly demonstrated for LMW-PAH-induced lung injury.

**Table 3:** Major communication media and their functions in the gut-lung axis.

Communication Medium	Representative Examples	Origin/Source	Main Functions in the Gut-Lung Axis	Potential Signaling Pathways Regulated	Research Source
Microbial metabolites	Short-chain fatty acids	*Bifidobacterium* spp.	Alleviate pulmonary inflammation	Induce reduction of pro-inflammatory cytokines; inhibit histone deacetylases (HDACs)	[70]
Microbial metabolites	Indole-3-lactic acid	Produced via tryptophan metabolism	Enhance intestinal barrier function; exert immunomodulatory effects	Activate the AhR signaling pathway; downregulate glycolysis, NF-κB and HIF signaling pathways, reducing epithelial CCL2/7 production	[93]
Immune cells	Regulatory T cells (Tregs)	Differentiated under the regulation of intestinal macrophages and dendritic cells	Inhibit inflammatory responses via cytokine secretion	Promote IgA secretion, thereby upregulating TGF-β expression to attenuate inflammatory responses	[62]
Immune cells	Th17 cells	Naive CD4+ T cells	Trigger inflammatory responses	Secrete cytokines including IL-17, IL-21, and IL-22	[94]
Inflammatory factors	TNF-α (Tumor Necrosis Factor-α)	Macrophages, B lymphocytes, T lymphocytes, natural killer cells, neutrophils, etc.	Trigger inflammatory responses	Activate the mitogen-activated protein kinase (MAPK) and c-Jun *N*-terminal kinase (JNK) signaling cascade, ultimately activating transcription factors NF-κB and Activator Protein 1 (AP-1)	[95]
Neurotransmitters	Acetylcholine	Vagus nerve efferent fibers	Protect intestinal barrier; attenuate inflammatory responses	Regulate cytokine release to reduce intestinal injury	[96]

**Table 4:** Mechanisms and potential outcomes related to LMW-PAH exposure and the gut-lung axis.

Mechanism	Main Manifestation	Potential Outcomes	Research Source
Gut Microbiota Dysbiosis	Intestinal inflammation, impaired digestive function, and immune dysfunction	Obesity, increased risk of chronic diseases, neurological disorders, skin diseases, and adverse effects on respiratory health	[107]
Gut Barrier Disruption	Intestinal inflammation, disruption of gut microbiota, and abdominal pain	Self-immune diseases, breast cancer, and aging	[108]
Immune Dysfunction	Inflammatory response	Acute respiratory distress syndrome and other lung injuries	[109]
Dysbiosis	Obesity, hypertension, hyperglycemia, and blood lipid abnormalities	Cardiovascular disease risk, type 2 diabetes, and fatty liver	[110]

**Table 5:** Interventional mechanisms of the gut-lung axis related to LMW-PAH exposure.

Interventional Mechanism	Specific Functions	Evidence Type	Research Source
Regulation of Gut Microbiota	Reduces inflammation, enhances lung function	Indirect (HCC mouse model)	[160]
Strengthening Intestinal Barrier	Alleviates inflammatory diseases, maintains gut homeostasis	Indirect (human RCT)	[159]
Immune Modulation	Enhances immune response, improves nutrient absorption, and reduces food intolerance	Indirect (review)	[161]
Metabolic Regulation	Antioxidant, anti-inflammatory effects, and supports cellular health	Indirect (review)	[162]

## Data Availability

No new data were created or analyzed in this study. Data sharing is not applicable to this article.
